# Projected incidence trends of need for long-term care in German men and women from 2011 to 2021

**DOI:** 10.3389/fepid.2023.1285893

**Published:** 2023-11-17

**Authors:** Sabrina Voß, Stephanie Knippschild, Luisa Haß, Thaddäus Tönnies, Ralph Brinks

**Affiliations:** ^1^ Faculty of Health, School of Medicine, Chair for Medical Biometry and Epidemiology, Witten/Herdecke University, Witten, Germany; ^2^ German Diabetes Center (DDZ), Leibniz Center for Diabetes Research at Heinrich-Heine-University, Institute for Biometrics and Epidemiology, Düsseldorf, Germany

**Keywords:** epidemiology, chronic conditions, illness-death model, aggregated data, partial differential equation, frailty, long-term care

## Abstract

**Background:**

The German Federal Statistical Office routinely collects and reports aggregated numbers of people in need of long-term care (NLTC) stratified by age and sex. Age- and sex-specific prevalence of NLTC from 2011 to 2021 is reported as well. One estimation of the incidence rate of NLTC based on the age- and sex-specific prevalence exists that did not explore possible trends in incidence [based on MRR (mortality rate ratio)], which is important for an adequate projection of the future number of people with NLTC.

**Objective:**

We aim to explore possible trends in age-specific incidence of NLTC in German men and women from 2011 to 2021 based on different scenarios about excess mortality (in terms of MRR).

**Methods:**

The incidence of NLTC was calculated based on an illness-death model and a related partial differential equation based on data from the Federal Statistical Office. Estimation of annual percent change (APC) of the incidence rate was conducted in eight scenarios.

**Results:**

There are consistent indications for trends in incidence for men and women aged 50–79 years with APC in incidence rate of more than +9% per year (up to nearly 19%). For ages 80+ the APC is between +0.4% and +12.5%. In all scenarios, women had higher age-specific APCs than men.

**Conclusion:**

We performed the first analysis of APC in the age- and sex-specific incidence rate of NLTC in Germany and revealed an increasing trend in the incidences. With these findings, a future prevalence of NLTC can be estimated which may exceed current prognoses.

## 1. Introduction

In the past decade (2011–2020) the prevalence of the need for long-term care (NLTC) increased from 3.4% to 5.9% of all German citizens with statutory health insurance (1). With the third “Care Strengthening Act” (“Pflegestärkungsgesetz”) (2) every German district (for example North Rhine-Westphalia) has to provide a projection of the future number of people in NLTC (3).

Current prognoses such as the prediction of the need for care from 2022 to 2070 (“Pflegevorausberechnung 2022 bis 2070”) from the Federal Statistical Office of the number of people with NLTC are based on the prevalence of NLTC only (4). Due to the fact that prevalence, incidence, and mortality interact (as described in the IDM and the associated differential equation), incidence and mortality should also be taken into account for the estimation of prevalence (5). Therefore, a projection based on the IDM should be preferred. To date, projections using the illness-death model (IDM) exist for other chronic conditions like for example type 1 diabetes (4). For NLTC, such a projection is still missing. Since the IDM describes the relations and dynamics between prevalence, incidence, and mortality, for a comprehensive image of future NLTC a projection based on the IDM and the related partial differential equation (PDE) needs to be added. The PDE describes the change of the age-specific prevalence at a specific point in time in terms of the incidence of NLTC, general mortality, and mortality rate ratio (MRR). The MRR is an epidemiological measure of excess mortality and is defined as the ratio of mortality rates of people with NLTC compared to those without NTLC. Accurate data about prevalence and general mortality are available from the Federal Statistical Office (6, 7), however, in a systematic literature search we found that estimates for the population-wide MRR in Germany have not been reported (8). As a consequence, the age-specific incidence rate of NLTC needs to be estimated.

Recently, the age-specific incidence rate of NLTC in Germany in 2015 was estimated based on the IDM from population-wide prevalence data for the years 2011–2019 using a PDE (8). As in (8) only a possible range of the incidence rates was reported, in this work, we consider scenarios about the MRR to gain an understanding of what possible trends in the age-specific incidence rate might look like. This work estimates the annual percentage change (APC) of the incidence rate of NTLC for men and women at ages 55, 65, 75, 85, and 95 years of age based on the IDM and justified assumptions about the MRR. Since the analysis was conducted (8), an additional year of prevalence data (for the year 2021) has been published, so the estimates of APCs in this work refer to the period 2011–2021. The aim of the work is the assessment of possible annual changes in the age-specific incidence rate of NLTC in order to be able to take these into account in a planned prognosis of NLTC prevalence in Germany.

## 2. Methods

### 2.1. Definition of NLTC

NLTC was determined as it is defined legally in Germany (in §14 Abs.1 SGB XI): A person in need of care is impaired in his or her independence or abilities due to health reasons and needs help as he or she is unable to compensate or manage physical, cognitive, or mental impairment or health-related burdens independently. Need of care has to last for at least 6 months with severity as is described in §15 SGB XI (4).

### 2.2. Data

The data used for calculations of incidence and APC in incidence based on an IDM was a combination of data that we collected in two databases. Data about NLTC was collected from (6), the Federal Statistical Office, which summarizes numbers about people in need of long-term care every two years. In our analysis, we used the information provided for the years 2011–2021. Mortality data were obtained from the Federal Statistical Office (7) with the assumption of a moderate development of birth rate, life expectancy, and migration (G2L2W2) collected for the 15th coordinated population projection for Germany.

### 2.3. Illness-death model and partial differential equation

The illness-death model describes the dynamics of a chronic condition with mortality rates and the incidence rate as transition intensities between 3 different states (no NLTC, NLTC, death). A related partial differential equation offers the opportunity to describe the prevalence depending on age *a* and calendar time *t*:

$$\left(\frac{\partial }{\partial a}+\;\frac{\partial }{\partial t}\right)p=(1-p)\;\left(i-\;m\frac{p(R-1)}{p(R-1)+1}\right)$$

This PDE can be transformed into the following equation to calculate the incidence of NLTC (8):

[1]. $$i=\frac{\left(\frac{\partial }{\partial a}+\;\frac{\partial }{\partial t}\right)p}{1-p}+m\;\frac{p(R-1)}{p(R-1)+1}$$

As in (8) age-specific prevalence data *p* and general mortality rates *m* stem from the official statistics of the Federal Statistical Office of Germany. Due to the lack of data about the MRR in Germany, scenarios need to be considered for estimating the incidence rate *i*. The systematic literature search presented in (8) found that the age-specific MRR lies in the range from 1.2 to 3.2. Based on empirical observations from chronic conditions (9, 10), it is assumed that the graph of the logarithmized MRR depends affine-linearly on age, i.e., that the graph of the log(MRR) over age is a straight line.

In accordance with (11) the remission rate (from NLTC back to no NLTC) was assumed to be zero.

### 2.4. Scenarios of MRR

The assumptions about the MRR for the eight considered scenarios are summarized in Table 1. In the first scenario (1: base case), the MRR starts at a value of 3.2 at age 50 and decreases to 1.2 at age 95 years. No trend of the MRR in time *t* over the period 2011–2021 is assumed in the base case scenario, which means that the APC of the MRR equals 0 in the first of the considered scenarios.

**Table 1:** Considered scenarios about the mortality rate ratios (MRR).

Scenario	MRR		Annual percent change (APC) of MRR
	At age 50	At age 95	
1 (base case)	3.2	1.2	0.0%
2	3.2	1.2	−2.5%
3	3.2	1.2	−5.0%
4	3.2	1.2	−10.0%
5	3.2	3.2	0.0%
6	3.2	3.2	−5.0%
7	1.2	1.2	0.0%
8	1.2	1.2	−5.0%

Scenarios 2–4 have identical values for the MRR at ages 50 (3.2) and 95 (1.2) as the base case. The difference between these scenarios and the base case is that we assume different APCs in the MRR from 2011 to 2021: −2.5% in scenario 2, −5.0% in scenario 3, and −10% in scenario 4. The value of −2.5% for the APC in scenario 2 was chosen based on the observation in Scandinavian registries of chronic conditions (10). This value reflects medical progress in treating people with chronic health conditions.

The higher values of −5.0% and −10.0% for the APCs in scenarios 3 and 4, respectively, have been chosen because in past years increasingly more people were reported to have NLTC. Possible reasons may be reimbursements from the statutory care insurance and a re-definition of NTLC in Germany in 2017. This may lead to a lowered MRR as the new system with 5 levels now assigns NLTC for persons that would not have been classified to a care level in the old system (NLTC assigned with fewer health restrictions). These persons potentially have lower mortality. However, a value of APC = −10% is considered as an extreme case only. It implies that the MRR halves every 6.58 years [log(0.5)/log(0.9) = 6.58 - APC = −5% implying a halving every 13.51 years [log(0.5)/log(0.95) = 13.51] and APC = −2.5% every 27.38 years [log(0.5)/log(0.975) = 27.38 respectively].

Scenarios 5 and 6 assume a constant MRR of 3.2 from 50 to 95 years of age with no APC (scenario 5) or an APC of −5.0% (scenario 6). Scenarios 7 and 8 also have constant (independent of age) values of MRR with a lower value of 1.2 for ages 50– 95 years. The difference between scenarios 7 and 8 lies in the expected APC of 0.0% (scenario 7) or −5.0% (scenario 8).

MRR values are limited to values greater or equal to 1 in all scenarios.

### 2.5. Statistical analysis

The APC in the age-specific incidence rates calculated with formula [1] was extracted with the following formula:

$$\begin{array}{rl} APC\;= & \;100\cdot \;\left\{{\left(\frac{i(2021,a)}{i(2011,a)}\right)}^{0.1}-1\right\}\mathrm{with}\;a\;\\ & \in \;\{55,65,75,85,95\} \end{array}.$$

with i(2011,a) and i(2021,a) being the age- and sex-specific incidence rates in 2011 and 2021 respectively. As the incidence rate is calculated using relations described in the IDM, the APC of *i* is calculated based on different scenarios of the APC in MRR (described in 2.4).

All calculations were carried out for men and women separately using the freely accessible, open-source statistical software R Version 4.1.0 (12). Data and source code for all results presented in this manuscript are available in the permanent, freely accessible online repository Zenodo (13).

## 3. Results

### 3.1. Estimated annual change in age-specific incidence rates

APC was calculated between 2011 and 2021 in eight different scenarios of MRR and APC of MRR and evaluated for the ages 55, 65, 75, 85, and 95 years as an example. The resulting estimated APCs in the incidence rates are shown in Table 2 and Supplementary Figure S1. Increasing incidences of NLTC from 2011 to 2021 were found for all ages and all scenarios. APC was decreasing with age, so higher values were seen at younger ages. The highest value of +18.7% was seen for men in scenarios 7 and 8 at age 55.

**Table 2:** Annual percent change (APC) of the incidence rate at specific ages for men and women during 2011 and 2021.

Scenario	Sex	APC of incidence rate at age				
		55 years	65 years	75 years	85 years	95 years
1	Male	17.7	15.7	12.4	8.6	3.0
	Female	18.3	15.6	14.0	12.0	8.0
2	Male	17.6	15.4	11.9	7.8	1.7
	Female	18.2	15.5	13.8	11.5	7.3
3	Male	17.5	15.2	11.5	7.3	1.7
	Female	18.2	15.4	13.6	11.3	7.3
4	Male	17.3	14.9	10.9	7.3	1.7
	Female	18.1	15.3	13.3	11.3	7.3
5	Male	17.5	14.8	10.8	6.7	2.0
	Female	18.2	15.3	13.0	10.1	5.6
6	Male	17.3	14.2	9.7	5.3	0.4
	Female	18.1	15.0	12.5	9.5	4.8
7	Male	18.7	17.1	13.6	9.1	3.0
	Female	18.6	16.1	14.6	12.5	8.0
8	Male	18.7	16.9	13.3	8.5	1.7
	Female	18.5	16.0	14.4	12.2	7.3

Differences in APC between sexes increase with age as well resulting in a bigger gap between APC for men and women at age 95 than at age 55. The biggest differences are seen at age 95. For example, APC in the base case at age 95 was +3.0% for men and +8.0% for women. The annual increase in the incidence of NLTC was higher for women in all scenarios (2.7 times higher in the base case scenario at age 95).

Overall, an increase in incidence (APC greater than +0.0%) of NLTC between 2011 and 2021 was found for all ages and both sexes in every scenario.

Additionally, calculated age-specific incidence rates of NLTC in 2021 are presented in Supplementary Table S1 for men and women separately.

## 4. Discussion

### 4.1. Summary

We estimated and explored possible trends in age-specific incidence of the need for long-term care in Germany. The analysis was stratified by sex for the years 2011–2021. The basis for the exploration of incidence trends was different scenarios about excess mortality (in terms of the mortality rate ratio). There were consistent indications for trends in incidence for men and women aged 50–79 years with APCs of more than +9% per year (up to nearly 19%). For ages 80+ the APCs in the considered scenarios is between +0.4% and +12.5%. In all scenarios, age-specific APCs for women are consistently higher than those for men. One reason may be that greater increases in prevalence were found in women than in men, both between years and between age groups (1, 14, 15).

### 4.2. Limitations

The current work has some limitations. The most important limitation of this work is the lack of empirical data about the incidence and mortality of people with NLTC. In principle, both epidemiological figures could empirically be estimated and reported. To overcome the lack of data about incidence, an analytical relation based on a PDE was used to indirectly estimate the change in the incidence rate over the period 2011–2021. To use the PDE approach, the mortality rate ratio of people with NLTC compared to people without NLTC is necessary. In a recent systematic literature search, these data have not been available for Germany (8). For this reason, different scenarios about the MRR had to be considered. By considering scenarios, we can only estimate a range of possible incidence trends.

However, definite statements on how incidence trends have been during the examined period from 2011 to 2021 are not possible at the time of this analysis. Given the huge burden of NLTC on affected individuals, family members, relatives, and societies, future research about the incidence and mortality of people in need of long-term care is urgently necessary.

In any of the considered scenarios, MRR values have been limited to values greater or equal to 1. An MRR lower than 1 would reflect the situation of NLTC being protective against mortality with the mortality rate of people with NLTC being lower than the rate for people without NTLC. While one might argue that people with NLTC may be monitored more frequently by nurses and other medically trained personnel, the recent systematic review (8) has not given any indication that NLTC is a protective factor against mortality. Moreover, the assumption that NLTC might be protective against mortality is a contradiction to the medical severity and enormous burden of the condition NLTC.

An additional limitation concerning the data basis is the change from 3 to 5 levels of care in Germany in 2017. This change may have affected the incidence and APC estimation (8). Due to this change in the definition of NLTC, the number of people in need of care has increased (in the first half of 2017 by 3.6% compared to the end of 2016). The new system with 5 care levels (since 2017) also takes cognitive restrictions into account instead of somatically-related restrictions only (before 2017) (15). An underestimation of future NLTC incidence rates is possible as people can be classified as NLTC easier and younger (and potentially longer) leading to a greater amount of people with NLTC. Moreover, it is also possible that the 2017 increase led to an overestimation of the changes in incidence. The exact direction and magnitude of the impact of this change on our APC estimation is unknown and could be analyzed in further studies.

Another limitation is that data from the COVID-19 pandemic is included as well which might have influenced our estimation of the incidence (trend) of NLTC. During the pandemic, mortality of nursing home residents increased from 25.6% to 28% (1). In addition to that, Tests for the presence of NLTC were temporarily not carried out or took place by telephone (14).

Despite the limitations mentioned above, the advantage of the presented work is that it is the basis for comprehensive projections of the future prevalence of NLTC in Germany, taking into account realistic increases in age-specific incidence.

### 4.3. Literature comparison

As far as the authors are aware, only one further estimation of the NLTC incidence in Germany exists for the year 2015. In contrast to the work presented here, that estimation did not consider the APC of the MRR resulting in an incidence estimation without APC (8). For this reason, we are the first to present the annual change in the age-specific incidence rate of long-term care in Germany. As (8) used the same data basis and the same definition of NLTC the results are comparable, with the advantage that we had the opportunity to add the additional year 2021. With our results and the results from (8) we now have the opportunity to estimate the future prevalence of NLTC taking into account the age-specific incidence rate with annual changes. As these estimations are based on German prevalence data that depend on the German definition of NLTC, they are not directly transferable to other (European) countries/regions.

## 5. Conclusion

We are the first to have calculated the annual percent change in the age-specific incidence for long-term care in Germany. The findings presented show an increase in the incidence rate with age and year for men as well as for women. These annual increases must be taken into account in projections to predict a realistic future burden of need for long-term care. The opportunity given in this work may lead to projected numbers of persons in long-term care that exceed current prognoses.

## Data Availability

Publicly available datasets were analyzed in this study. This data can be found here: The dataset analyzed during this study is available from the free and open repository Zenodo with digital object identifier (DOI) 10.5281/zenodo.7849193. The underlying prevalence data had been accessed from the “Pflegestatistik—Pflege im Rahmen der Pflegeversicherung Deutschlandergebnisse“ from the years 2011–2021 (see https://www.statistischebibliothek.de/mir/receive/DESerie_mods_00000940). Mortality data were obtained from the Federal Statistical Office https://service.destatis.de/bevoelkerungspyramide/ with the assumption of a moderate development of birth rate, life expectancy and migration (G2L2W2).
