# Supplementary material for: Projected incidence trends of need for long-term care in German men and women from 2011 to 2021
Source: Front Epidemiol. 2023 Nov 17;3:1285893. doi: 10.3389/fepid.2023.1285893 (PMC10910887; doi:10.3389/fepid.2023.1285893)
Supplement: Supplementary file 1 [file Table1.docx]

Supplementary Material

Possible incidence trends of need for long-term care in German men and women from 2011 to 2021

Sabrina Tulka*, Stephanie Knippschild, Luisa Haß, Thaddäus Tönnies^1^, Ralph Brinks *Correspondence: Sabrina Tulka: [sabrina.tulka@uni-wh.de](mailto:sabrina.tulka@uni-wh.de)

1 Supplementary Table

**Supplementary Table 1.**

Calculated incidence rate at specific ages for men and women in 2021 in Germany.

| Scenario | Sex | APC of incidence rate at age | | | | |
| --- | --- | --- | --- | --- | --- | --- |
|  |  | 55 years | 65 years | 75 years | 85 years | 95 years |
| 1 | Male | 0.004 | 0.010 | 0.030 | 0.101 | 0.237 |
|  | Female | 0.004 | 0.011 | 0.041 | 0.193 | 0.488 |
| 2 | Male | 0.004 | 0.010 | 0.029 | 0.093 | 0.208 |
|  | Female | 0.004 | 0.011 | 0.040 | 0.186 | 0.457 |
| 3 | Male | 0.004 | 0.010 | 0.028 | 0.089 | 0.208 |
|  | Female | 0.004 | 0.011 | 0.040 | 0.182 | 0.457 |
| 4 | Male | 0.004 | 0.010 | 0.027 | 0.089 | 0.208 |
|  | Female | 0.004 | 0.011 | 0.039 | 0.182 | 0.457 |
| 5 | Male | 0.005 | 0.011 | 0.033 | 0.124 | 0.347 |
|  | Female | 0.004 | 0.011 | 0.043 | 0.211 | 0.593 |
| 6 | Male | 0.004 | 0.010 | 0.030 | 0.108 | 0.299 |
|  | Female | 0.004 | 0.011 | 0.041 | 0.199 | 0.550 |
| 7 | Male | 0.004 | 0.010 | 0.028 | 0.094 | 0.237 |
|  | Female | 0.004 | 0.011 | 0.040 | 0.187 | 0.488 |
| 8 | Male | 0.004 | 0.010 | 0.027 | 0.089 | 0.208 |
|  | Female | 0.004 | 0.011 | 0.039 | 0.182 | 0.457 |
