# Supplementary figures and images for: Projected incidence trends of need for long-term care in German men and women from 2011 to 2021
Source: Front Epidemiol. 2023 Nov 17;3:1285893. doi: 10.3389/fepid.2023.1285893 (PMC10910887; doi:10.3389/fepid.2023.1285893)

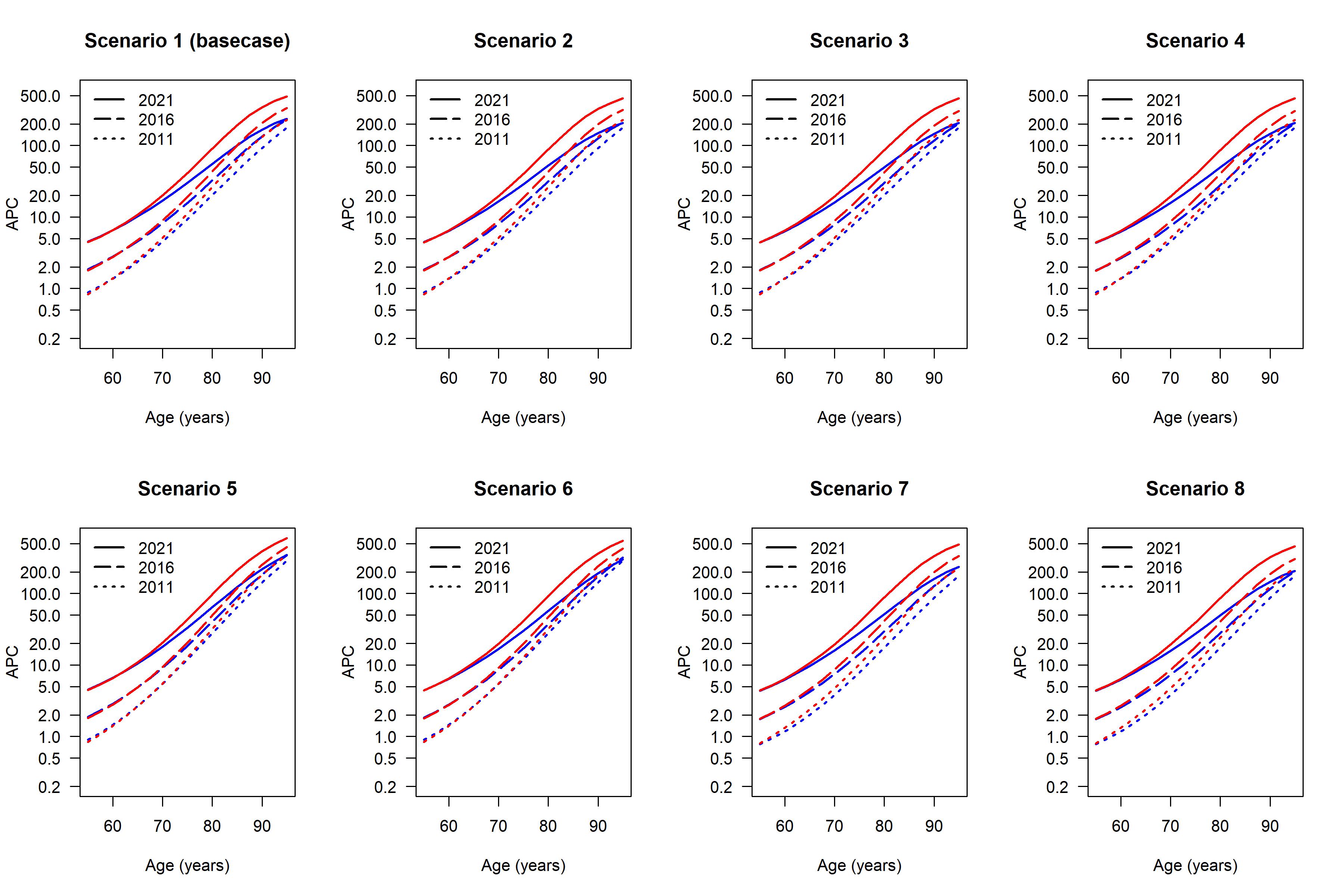

Supplement: Supplementary file 2 [file Image1.jpeg]
